# Tojapride prevents CaSR‐mediated NLRP3 inflammasome activation in oesophageal epithelium irritated by acidic bile salts

**DOI:** 10.1111/jcmm.14631

**Published:** 2019-12-20

**Authors:** Xiao‐Lan Yin, Hao‐Meng Wu, Bei‐Huang Zhang, Ning‐Wei Zhu, Ting Chen, Xiang‐Xue Ma, Li‐Ying Zhang, Lin Lv, Min Zhang, Feng‐Yun Wang, Xu‐Dong Tang

**Affiliations:** ^1^ Department of Gastroenterology China Academy of Chinese Medical Sciences Xiyuan Hospital Beijing China; ^2^ Department of Gastroenterology, Guangzhou Higher Education Mega Center The Second Affiliated Hospital of Guangzhou University of Chinese Medicine, Xiao‐gu‐wei Jie Guangzhou China; ^3^ Department of Pharmacy Zhejiang Pharmaceutical College Ningbo China

**Keywords:** CaSR, NLRP3, oesophageal epithelium, reflux oesophagitis, Tojapride

## Abstract

Impairment of the oesophageal epithelium in patients with reflux oesophagitis (RE) is a cytokine‐mediated injury rather than a chemical burn. The present study was conducted to explore CaSR/NLRP3 inflammasome pathway activation and cytokines IL‐1β and IL‐18 release in oesophageal epithelia injured by refluxates and the effects of Tojapride on that signal regulation. Using a modified RE rat model with Tojapride administration and Tojapride‐pretreated SV40‐immortalized human oesophageal epithelial cells (HET‐1A) exposed to acidic bile salts pretreated with Tojapride, we evaluated the therapeutic effects of Tojapride on oesophageal epithelial barrier function, the expression of CaSR/NLRP3 inflammasome pathway‐related proteins and the release of downstream cytokines in response to acidic bile salt irritation. In vivo, Tojapride treatment ameliorated the general condition and pathological lesions of the oesophageal epithelium in modified RE rats. In addition, Tojapride effectively blocked the CaSR‐mediated NLRP3 inflammasome activation in modified RE rats. In vitro, Tojapride treatment can reverse the harmful effect of acidic bile salts, which reduced transepithelial electrical resistance (TEER), up‐regulated the CaSR‐mediated NLRP3 inflammasome pathway and increased caspase‐1 activity, LDH release and cytokines secretion. Taken together, these data show that Tojapride can prevent CaSR‐mediated NLRP3 inflammasome activation and alleviate oesophageal epithelial injury induced by acidic bile salt exposure.

## 1 INTRODUCTION

Reflux oesophagitis (RE) is caused by the excessive backflow of gastric/duodenal contents into the oesophagus, which induces inflammatory lesions and ulceration of the oesophageal mucosa, finally causing problematic symptoms and/or complications. RE is one of the major phenotypic manifestations of gastroesophageal reflux disease (GERD). Epidemiological estimates indicate a 10%‐20% prevalence of RE in Western countries, whereas the prevalence is <5% in Asian countries.^1^ RE represents a complication of GERD in approximately 30% of cases.^2^

In the past, RE was generally regarded as a chemical injury of the oesophageal epithelium caused by hydrochloric acid and pepsin. However, RE develops through a cytokine‐mediated injury rather than a chemical injury and progresses to the deep layers of the epithelium. In 2009, a study first suggested this new paradigm of RE pathogenesis.^3^ A recent clinical trial further validated this hypothesis by interrupting proton‐pump inhibitor (PPI) therapy in 12 patients with RE and examining inflammation in oesophageal biopsies,the inflammation was found to be predominantly associated with T lymphocytes with few infiltrating neutrophils and eosinophils, and notably, none of the biopsies showed a loss of surface cells.^4^ Typical oesophageal morphological abnormalities of RE are mainly characterized by basal cell hyperplasia and elongation of connective tissue papillae attributed to acid/alkali‐associated injury of the epithelial cells^5^ As mentioned above, studying the molecular pathway of RE injury is of fundamental importance because it offers insight into the pathogenesis of oesophageal mucosal inflammatory injury.

Calcium‐sensing receptor (CaSR) is a G protein‐coupled receptor that is widely expressed in intact polarized epithelia along the entire gastrointestinal tract to stimulate epithelial cell differentiation^6^ and regulate gastrointestinal immunity.^7^, ^8^ Notably, depending on the cell type, CaSR also participates in the regulation of cell proliferation via activatory or inhibitory effects.^9^, ^10^, ^11^ Up‐regulation of CaSR in HET‐1A cells can accelerate the secretion of the cytokine IL‐8^12^ which is in accordance with research showing that after brief exposure to acidic bile salts, human oesophageal cells do not die but secrete specific cytokines.^3^

The nucleotide‐binding and oligomerization domain‐like receptor family pyrin domain containing 3 (NLRP3) inflammasome is a signalling platform of the immune system.^13^ Specifically, aberrant overactivation of the NLRP3 inflammasome has recently been identified in Barrett's epithelial cells.^14^ Activation of the NLRP3 inflammasome is thought to consist of priming and assembly. The priming signal is usually induced by the Toll‐like receptor (TLR)/nuclear factor‐kappa B (NF‐κB) pathway, which up‐regulates the expression of NLRP3, procaspase‐1 and pro‐IL‐1β, the levels of which are otherwise relatively low in the cytoplasm. The NLRP3 inflammasome is a multiprotein complex that consists of NLRP3, apoptosis‐associated speck‐like protein containing a caspase recruitment domain (ASC) and procaspase‐1. Caspase‐1, which is derived from its precursor protein, is an inflammatory caspase that catalyses the proteolytic cleavage of pro‐IL‐1β to its mature, active forms and then initiates a inflammatory cell death, that is pyroptosis.^15^ A variety of molecular mechanisms have been confirmed in the activation of NLRP3 inflammation.^16^ Lipopolysaccharide (LPS) is a gram‐negative bacterial cell wall component that functions as a pathogen‐associated molecular pattern (PAMP) and up‐regulates the expression of TLR4 for inflammation priming.^17^ In the classic two signal activation (priming and activation) of NLRP3 inflammasome formation in various cell types, NF‐κB activation and nuclear translocation in response to TLR4 signalling are postulated to be the initial event in NLRP3 and pro‐IL‐1β transcription,^18^, ^19^ whereas LPS‐induced TLR4 signalling both primes and activates the NLRP3 inflammasome in human Barrett's epithelial cells.^14^ Geun‐Shik Lee et al first identified that Ca^2+^ or other CaSR agonists activate the NLRP3 inflammasome in LPS‐primed mouse bone marrow‐derived macrophages (BMDMs).^20^ Furthermore, Wenxiu Liu et al found that the CaSR/NLRP3 inflammasome pathway plays an essential role in M1, but not M2 macrophages in the process of cardiac inflammation and remodelling.^21^ Hence, the postulated CaSR‐mediated NLPR3 inflammasome activation is mainly present in the immune cell types such as THP‐1 cells and BMDMs. The role of CaSR/NLRP3 inflammasome signalling in epithelial cells is unclear.

Tojapride, a formula granule of traditional Chinese medicine (TCM), is composed of *Perilla frutescens, Cyperus rotundus and Fructus aurantii,* among others. High‐performance liquid chromatography (HPLC) has revealed that Tojapride contains more than 25.8 mg/g naringin and 20.1 mg/g neohesperidin (the data have not been made public). Currently, Tojapride is used as a regular, commercial, patented drug at the China Academy of Chinese Medical Sciences Xiyuan Hospital. Our previous randomized controlled trial (RCT) demonstrated that Tojapride effectively alleviated the symptoms of acid reflux and heartburn in patients with GERD.^22^ Modern pharmacological studies have identified the anti‐inflammatory effect of several individual Chinese herbs within Tojapride.^23^, ^24^, ^25^, ^26^

Psychological factors have drawn much attention in the pathogenesis of GERD and have a negative impact on quality of life (QoL).^27^ A recent epidemiological survey from China has further demonstrated that the incidence of GERD was correlated with anxiety and depression, and the QoL of patients was significantly reduced.^28^ According to the theory of TCM, Tojapride is effective in smoothing the liver and regulating gastric functions and be applied to the GERD patients with liver‐gastric disharmony syndrome, which is similar to that of GERD patients with psychological problems. In the present study, we adopted a modified RE rat model, in which the oesophagus was directly connected to the duodenum with a well‐preserved stomach, in combination with psychological irritation to investigate the expression of CaSR and the NLRP3 inflammasome in the oesophageal epithelium and the pharmacological efficacy of Tojapride. Furthermore, we explored the expression of the CaSR/NLRP3 inflammasome in acidic bile salt‐stimulated HET‐1A cells and whether Tojapride treatment might inhibit that expression.

## 2 MATERIALS AND METHODS

### 2.1 Establishment of the RE rat model

Eight‐week‐old male Sprague Dawley rats (Huafukang Bioscience Co. Inc) (No. 11401300049714), weighing 220 ± 20 g, were housed under standard laboratory conditions (room temperature, 25 ± 1°C; relative humidity, 65 ± 5%; regular air change; and a 12 hour light/dark cycle) and supplied with tap water and standard diet ad libitum. The rats were allowed to acclimate for 7 days before surgical intervention and were then was subjected to oesophagoduodenostomy.^29^ A total of 110 rats were operated on, and 12 out of 120 rats were randomly selected as sham group that undergone a simple celiotomy. Fasting for 24 hours postoperatively, the rats gradually returned to normal diet. All experimental procedures were approved by the Ethics Review Committee for Animal Experimentation of Xiyuan Hospital, China Academy of Chinese Medical Sciences, PR China.

### 2.2 Establishment of the modified RE rat model with tail clamp simulation

Two weeks postoperatively, the rats were housed 6 per cage with tail clamp stimulation to induce anger and fighting. The second third of each rat's tail was clamped with an iron clip for 20 minutes, and the clamp position was changed at 5‐minute intervals. The duration of daily stimulation was 45 minutes and lasted for 1 week.

### 2.3 Grouping and drug administration

**Figure 1:**
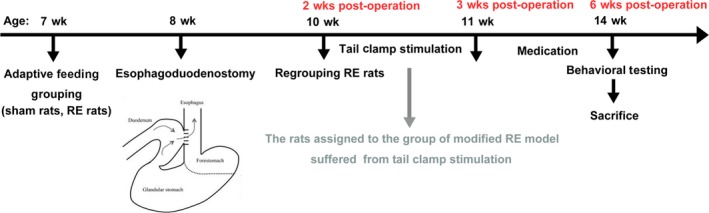
Timeline of model establishment, grouping and gastric administration in animal experiments. The drawing of oesophagoduodenostomy is from Savarino et al^1^

Three weeks postoperatively, excluding the sham group, the remaining rats were randomized into seven groups according to the model and treatment of each group, namely the RE model group, the modified RE model group, low‐, medium‐ and high‐dose Tojapride (ie Tojapride‐L, Tojapride‐M and Tojapride‐H) groups, the omeprazole group and the combination of Tojapride and omeprazole group. Administration was started 3 weeks postoperatively and maintained for 3 weeks. The flow of the animal experiments is shown in Figure 1.

Tojapride was intragastrically administered at dosages of 5.73 g/kg/d (Tojapride‐L), 11.46 g/kg/d (Tojapride‐M) and 22.92 g/kg/d (Tojapride‐H) in three model groups. Omeprazole was intragastrically administered at a dosage of 4.17 mg/kg/d, Tojapride combined with omeprazole was intragastrically administered at dosages of 11.46 g/kg/d Tojapride‐M and 4.17 mg/kg/d omeprazole (Tojapride‐omeprazole), and distilled water was administered in both sham rats and model rats groups.

### 2.4 Open‐field test

The experiments were performed in a relatively quiet environment without obvious interference. The rats were placed individually in the middle of a wooden open‐field apparatus with 100 cm length, 100 cm width and 40 cm height. Twenty‐five squares were delineated on the ground, nine squares were in the middle field, and 16 squares were in the peripheral region. The activity of each rat over a 5‐minute period was video‐recorded by a camera over the centre of the open‐field ground. The distance covered, duration each rat spent in the whole region and the number of times the rat crossed between squares were measured.^30^ To exclude batch variation, rats treated with distilled water were tested at irregular intervals between drug‐treated rats. The open‐field apparatus was carefully cleaned with 70% ethanol after each trial.

### 2.5 Gastric fluid collection and detection

Twelve hours before killing, the rats were underwent a celiotomy, and the pylorus was ligated with a 3‐0 silk suture. Gastric juice was collected in a centrifuge tube and centrifuged at 3000 *g* for 10 minutes at 4°C. The pH value of the supernatant was measured using a pH meter (Mettler‐Toledo Delta 320), and the amounts of pepsin, pepsinogen and bile acid were measured by an enzyme‐linked immunosorbent assays (ELISA) kit (BioMart) per the manufacturer's instructions.

### 2.6 Haematoxylin and eosin (HE) staining and pathological evaluation

HE staining was performed on paraffin‐embedded oesophageal section (5 μm). The embedded section was deparaffinized, hydrated and stained with HE. The stained sections were subsequently observed under an optical microscope.

### 2.7 Quantitative real‐time polymerase chain reaction (qPCR) analysis

Oesophageal strips were placed in TRIzol and sonicated. Total RNA was isolated from the tissues treated with chloroform, precipitated with isopropyl alcohol and resuspended in diethyl pyrocarbonate (DEPC)‐treated water. cDNA was reverse transcribed from total RNA by a FastQuant RT kit (TIANGEN BioMart). CaSR and NLRP3 mRNA expressions were determined by a SYBR Green PCR Master Mix Kit (Applied Biosystems, Thermo Fisher Scientific) performed with an ABI 7900 Fast Real‐Time PCR System. The reaction was carried out in a 20 μL final volume with an initial denaturation at 95℃ for 5 minutes, followed by 40 temperature cycles of 95°C for 30 seconds, 55°C for 30 seconds and 72°C for 35 seconds. The primers used were as follows: CaSR, forward primer 5' ACCTCCTGCTCTTCTCCCTA 3' and reverse primer 5' GTGGAAGCTGGTGGGTATCT 3'; NLRP3, forward primer 5' TGTCAGGATCTCGCATTGGT 3' and reverse primer 5' ACAGTGAAGTAAGGCCGGAA 3'; NLRC4, forward primer 5' ATCAGTCTGCCCAATCTGCT 3' and reverse primer 5' ACCGTGTCCTGCTAAATCCA 3'; NLRP1A, forward primer 5' TGTGCCAAGGTAGAACGGAT 3' and reverse primer 5' TTTGGGCATGTGAATGGGTG 3' and AIM2, forward primer 5' GGAGACAGCCAAGAGTGACT 3' and reverse primer 5' CCCTGTTCCCTGAGTGTTCT 3'. In addition, the primer of forward primer 5' CCATGGAGAAGGCTGGG 3' and reverse primer 5' CAAAGTTGTCATGGATGACC 3' was used for the detection of GAPDH, which was used as a reference gene.

### 2.8 Western blot analysis

Oesophageal strips or cultured cells were collected to extract the protein with RIPA lysis buffer (Beyotime Biotech), which contained 1 mmol/L final concentration of phenylmethanesulfonyl fluoride (PMSF). After complete lysis, the samples were centrifuged at 10 000 *g* for 5 minutes at 4°C to precipitate the tissue debris. The supernatants were used to measure the protein concentration by a BCA Protein Assay Kit (Beyotime Biotech). The proteins were electrophoresed in 10% SDS‐PAGE gels and then transferred to PVDF membranes. After blocking with 5% skim milk for 1 hour at room temperature, the membranes were incubated with the following primary antibodies: CaSR (1:200, ab19347, Abcam), NLRP3 (1:100, ab214185, Abcam), ASC (1:100, sc‐22514‐R, Santa Cruz), Caspase‐1 p20 (1:100, sc‐398715, Santa Cruz) and IL‐1β (1:100, sc‐32294, Santa Cruz) at 4°C overnight. The membrane was washed with TBST and incubated with secondary antibodies for 2 hours at room temperature. Protein bands were visualized on the membrane with a Gel Imaging System, and the protein bands were quantified with ImageJ software.

### 2.9 Immunohistochemistry

Immunohistochemistry was performed with paraffin‐embedded sections. After deparaffinization, rehydration and blocking, the sections were incubated with the following primary antibodies: CaSR (1:100, ab19347, Abcam); NLRP3 (1:100, ab4207, Abcam); and ASC (1:100, sc‐22514‐R, Santa Cruz). After washing with PBS, the sections used to detect CaSR through enzyme‐linked immunohistochemistry were incubated with secondary biotinylated antibodies and detected using a streptavidin‐peroxidase (SP)–conjugated system by UltraSensitive SP kit (Maxim). Mounted sections were subsequently observed under an optical microscope. In addition, the sections used to detect NLRP3‐ASC colocalization through immunofluorescence were incubated with FITC‐ or Cy3‐labelled secondary antibodies (Beyotime Biotech). Images were acquired by a confocal laser scanning microscope (FV1000, Olympus).

### 2.10 Cell culture

HET‐1A cells obtained from American Type Culture Collection (ATCC) were cultured in bronchial epithelial basal medium (BEBM; Lonza) supplemented with 100 U/mL penicillin‐streptomycin solution (Invitrogen). The cells were cultured in a humidified incubator containing 5% carbon dioxide (CO_2_).

### 2.11 Acid and bile salt exposure

Cells were exposed to acid, bile salts and acidic bile salt media. The media have been described by Xiaofang Huo.^31^, ^32^ When the cells reached 75%‐80% confluence, they were treated with the different media for three times per day at 10 minutes per treatment.

### 2.12 TEER

The TEER of the oesophageal epithelial monolayer was measured using a chamber system. HET‐1A cells, which were cultured in transwell plates, were mounted on a perfusion slot, bathed with Kreb's solution (pH 7.4) on both the apical and basal sides and maintained in a mixed gas flow (O_2_/CO_2_ = 95:5). A temperature cycle water bath was used to ensure a stable temperature of 37°C. After the multichannel current clamp was started and the resistance of the cells was established and stabilized, we recorded the TEER for 40 minutes.

### 2.13 Measurement of caspase‐1 activity

The activity of caspase‐1 was detected using a Caspase 1 Assay kit (ab39412, Abcam) per the manufacturer's instructions. All assays were performed in triplicate in three independent experiments.

### 2.14 Measurement of LDH

LDH release from the pyroptotic cells was detected using a LDH Cytotoxicity Assay Kit (Dojindo) per the manufacturer's instructions. All assays were performed in triplicate in three independent experiments.

### 2.15 Elisas for IL‐1β and IL‐18

Supernatants from HET‐1A cell cultures were collected and centrifuged to remove cell debris. The concentration of IL‐1β and IL‐18 in the culture supernatants was determined by using a cytokine‐specific ELISA kit (Biomart) per the manufacturer's instructions. All assays were performed in triplicate in three independent experiments.

## 3 RESULTS

### 3.1 Modified RE rats exhibited anxiety‐related behaviour and Tojapride ameliorated the anxiety of modified RE rats

**Figure 2:**
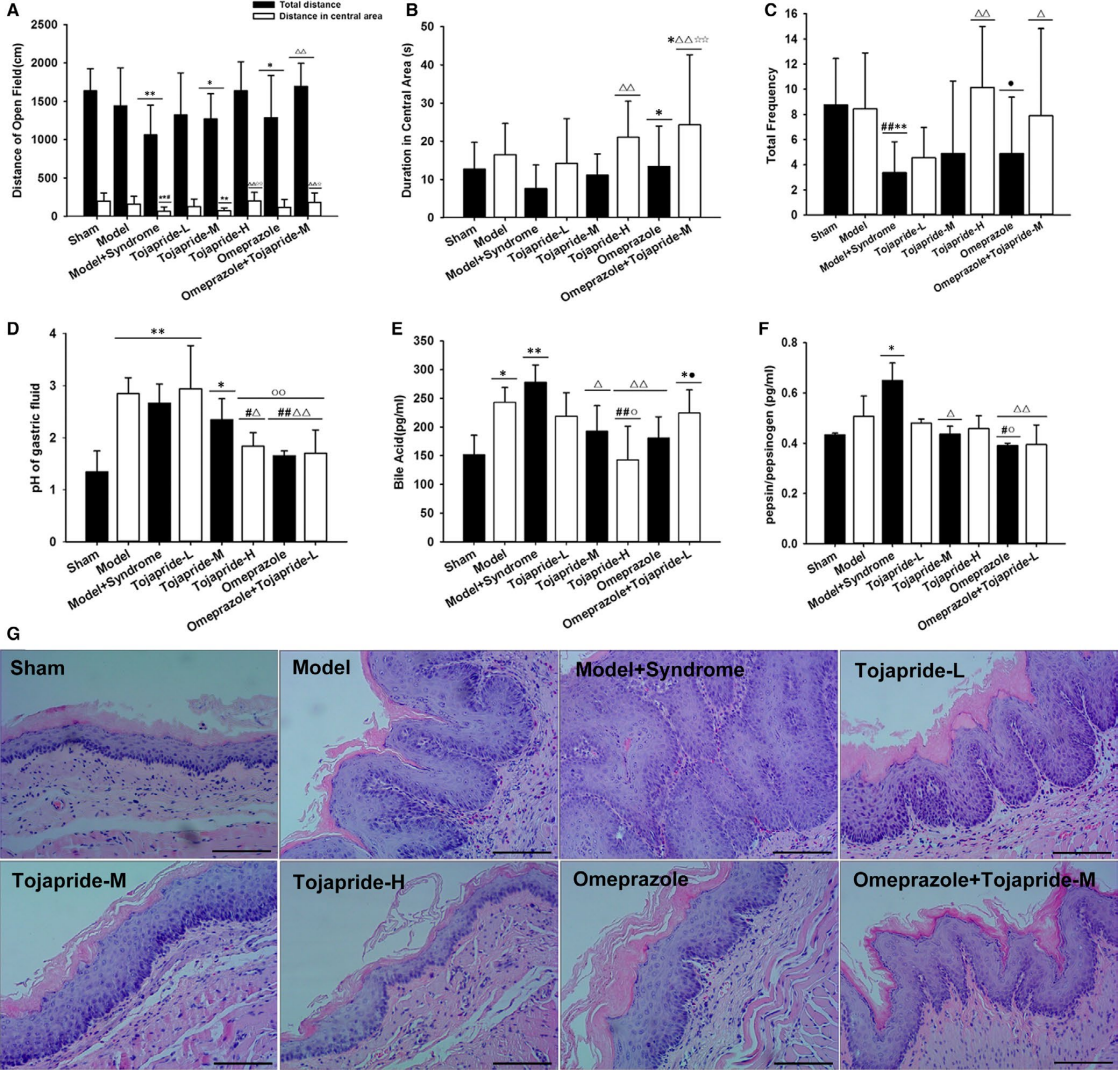
Effects of Tojapride on anxiety‐related behaviour, damage‐inducing factors of the refluxates and pathological lesions of the oesophagus in modified RE rats. Open‐field test for (A) total distance and distance travelled in the central area, (B) time spent in the centre and (C) the number of times when the rat crossed the squares. Gastric fluid detection for (D) pH of gastric fluid, (E) bile acid and (F) pepsin to pepsinogen ratio. G, HE staining of the oesophagus in each group. Bar graphs represent the means ± SD. *P < .05 and **P < .01, compared with the control group (sham rats); #*P* < .05 and ##*P* < .01, compared with the model group (RE rats); △*P* < .05 and △△*P* < .01, compared with the model‐syndrome group (modified RE rats); ○*P* < .05 and ○○*P* < .01, compared with the Tojapride‐L–treated group; ☆*P* < .05 and ☆☆*P* < .01, compared with the Tojapride‐M–treated group; ●*P* < .05 and ●●*P* < .01, compared with the Tojapride‐H–treated group; ☆*P* < .05 and ☆☆*P* < .01, compared with the Tojapride‐omeprazole–treated group

Thirty‐eight of 120 rats died by week 6 after surgery (survival rates: 62%). First, to determine whether the modified RE rats were predisposed to anxiety state, we used an open‐field test to evaluate anxiety‐related and autonomous behaviours. We found that modified RE rats explored the centre of the open field less than the sham rats and RE rats (Figure2A). Although there was no significant difference among the sham rats, RE rats and modified RE rats (Figure2B), modified RE rats were more immobile with shorter total distances and fewer lattice crossings (Figure2A,C). After three weeks of gavage, Tojapride‐H and Tojapride‐omeprazole treatment significantly increased the distance covered and duration spent in the central area and elevated the frequency of lattice crossings by the rats (Figure2A,B,C).

### 3.2 Modified RE rats exhibited reflux of gastric and duodenal contents, and the Tojapride decreased the pH value, bile acid and pepsin of the collected gastric fluid of modified RE rats

Oesophageal epithelial exposure to gastric and bile acids or pepsin is the predominant outcome of mixed reflux.^33^ We examined the pH value, bile acid concentration and relative pepsin concentration in the collected gastric fluid of rats in each group. Because the lumen of the oesophagus and duodenum were interlinked, the rats suffered from oesophagoduodenostomy and had higher pH values and bile acid concentrations than the sham rats (Figure 2D,E). In addition, modified RE rats exhibited an increased pepsin to pepsinogen ratio, which was further increased in RE rats; however, there was no significant difference between RE rats and sham rats (Figure 2F). Compared with modified RE rats, rats administered Tojapride‐H, omeprazole and Tojapride‐omeprazole had significantly decreased gastric fluid pH. Tojapride‐M, Tojapride‐H and omeprazole treatment significantly decreased the bile acid concentration, and Tojapride‐M, omeprazole and Tojapride‐omeprazole treatment significantly decreased the ratio of pepsin to pepsinogen (Figure 2D,E,F).

### 3.3 Modified RE rats exhibited severe pathological lesions of the oesophageal epithelium, and Tojapride treatment partially reversed the pathological changes

Basal cell hyperplasia and papillary elongation were observed in the oesophageal epithelium of RE rats, and these pathological changes peaked to a degree in modified RE rats. Compared with the markedly thickened epithelium of modified RE rats, the epithelium of rats treated with both Tojapride and omeprazole displayed reversed basal cell and papillary hyperplasia, but not to the extent observed in the epithelium of the sham rats (Figure2G).

### 3.4 Modified RE rats exhibited up‐regulation of the CaSR‐mediated NLRP3 inflammasome signalling pathway, and Tojapride effectively blocked CaSR‐mediated NLRP3 inflammasome activation

**Figure 3:**
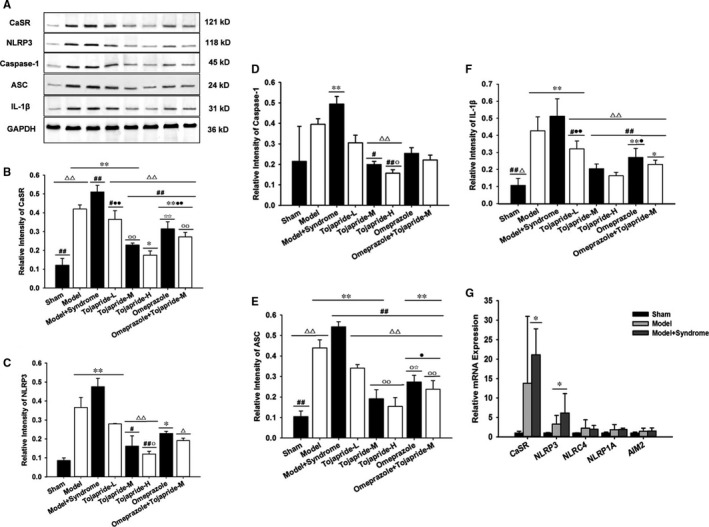
Inhibitory effects of Tojapride on the up‐regulated CaSR‐mediated NLRP3 inflammation activation in modified RE rats. Western blot for the expression of (A, B) CaSR, (A, C) NLRP3, (A, D) caspase‐1, (A, E) ASC and (A, F) IL‐1β in each group. G, mRNA expression of CaSR and inflammasome PRRs (NLRP3, NLRC4, NLRP1A and AIM2) within sham rats, pure RE rats and modified RE rats. Bar graphs represent the means ± SD. *P < .05 and **P < .01, compared with the control group (sham rats); #*P* < .05 and ##*P* < .01, compared with the model group (RE rats); △*P* < .05 and △△*P* < .01, compared with the model‐syndrome group (modified RE rats); ○*P* < .05 and ○○*P* < .01, compared with the Tojapride‐L–treated group; ☆*P* < .05 and ☆☆*P* < .01, compared with the Tojapride‐M–treated group; ●*P* < .05 and ●●*P* < .01, compared with the Tojapride‐H–treated group; and ☆*P* < .05 and ☆☆*P* < .01, compared with the Tojapride‐omeprazole–treated group

**Figure 4:**
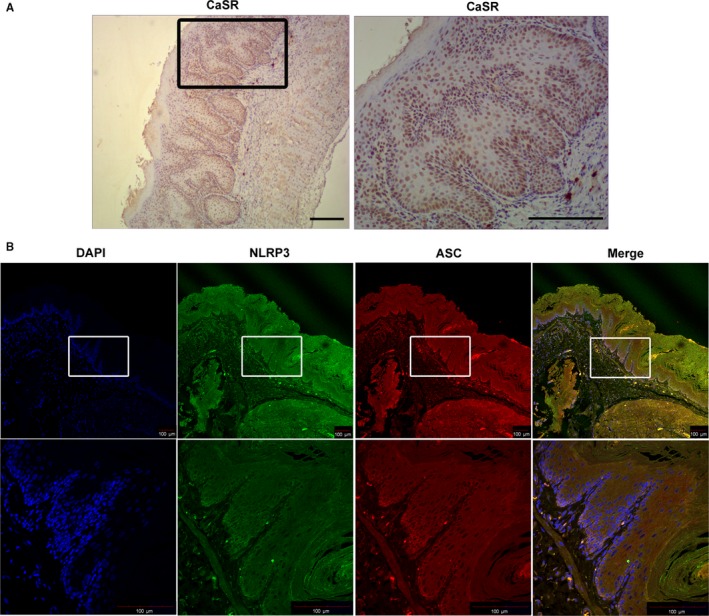
CaSR expression and colocalization of NLRP3‐ASC in the oesophagus of modified RE rats. A, Enzyme‐linked immunohistochemistry in paraffin‐embedded sections of oesophageal tissue using a mouse monoclonal CaSR antibody. Scale bar: 200 μm. B, Immunofluorescence in paraffin‐embedded sections of oesophageal tissue using goat polyclonal NLRP3 (green) and rabbit polyclonal ASC (red) antibodies. Scale bar: 100 μm. The yellow area in the basal cells of the epithelium indicates the colocalization of the green and red channels

To assess whether the CaSR/NLRP3 inflammasome signalling pathway is up‐regulated in the impaired oesophagus, we used qPCR and Western blot techniques to test the expression of pathway‐related proteins. We found that CaSR, NLRP3, caspase‐1, ASC and IL‐1β were significantly up‐regulated in the oesophagus of modified RE rats (Figure 3). Immunolocalization studies showed CaSR to be mainly expressed in the basal and suprabasal layers, and NLRP3‐ASC was localized in the basal layer and lamina propria (Figure 4). Moreover, NLRP3 was the only pattern recognition receptor (PRR) expressed in the oesophagus of both modified RE rats and pure RE rats to a greater extent than that in sham rats at the mRNA level. There was no significant difference in NLRC4, NLRP1A and AIM2 expression between RE rats and sham rats (Figure 3G). Compared with modified RE rats, Tojapride‐M– and Tojapride‐H–treated rats had significantly reduced the expression levels of CaSR, NLRP3, caspase‐1, ASC and IL‐1β, and Tojapride‐L–, omeprazole– and Tojapride‐omeprazole–treated rats had reduced expression levels of CaSR, ASC and IL‐1β. Within the medication groups, the Tojapride‐H group had a lower expression of CaSR, NLRP3, caspase‐1, ASC and IL‐1β than the Tojapride‐L group (Figure 3).

### 3.5 LPS and acidic bile salts treatment reduced the TEER in HET‐1A monolayer cells, and Tojapride attenuated acid bile salt‐induced oesophageal epithelial barrier damage

**Figure 5:**
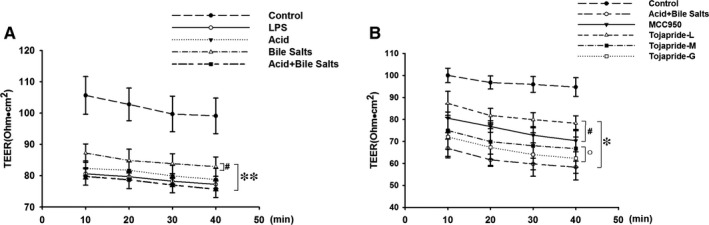
Treatment of HET‐1A monolayer cells with acidic bile salts reduced the TEER, and Tojapride treatment attenuated the oesophageal epithelial barrier damage induced by acidic bile salts. A, TEER of HET‐1A monolayer cells treated with LPS, acid, bile salts and acidic bile salts. B, The effect of Tojapride on HET‐1A monolayer cells treated with acidic bile salts. Data are shown as means ± SD. *P < .05 and **P < .05, compared with the control group; #*P* < .05 and ##*P* < .01, compared with the acidic bile salt‐treated group; and ○*P* < .05, compared with Tojapride‐L–treated group

To investigate whether the exposure of HET‐1A monolayer cells to an acidic bile salt cocktail affects oesophageal epithelial barrier function, we tested the TEER of HET‐1A cells to assess the effect of LPS, acid, bile salt and acidic bile salts. Remarkably, the TEER of HET‐1A monolayer cells significantly declined after treatment with LPS, acid, bile salt and acidic bile salt media. Furthermore, HET‐1A monolayer cells treated with acidic bile salt media presented lower TEER values than those treated with pure bile salt media (Figure 5A). Pretreatment with Tojapride‐L and MCC950, a specific small‐molecule NLRP3 inhibitor, blocked acidic bile salt‐induced TEER decline, but not completely (Figure 5B).

### 3.6 Exposure of HET‐1A cells to acid and bile salts accelerates CaSR‐mediated NLRP3 inflammasome activation, and Tojapride down‐regulates the CaSR‐mediated NLRP3 inflammasome signalling pathway induced by acidic bile salts in HET‐1A cells

**Figure 6:**
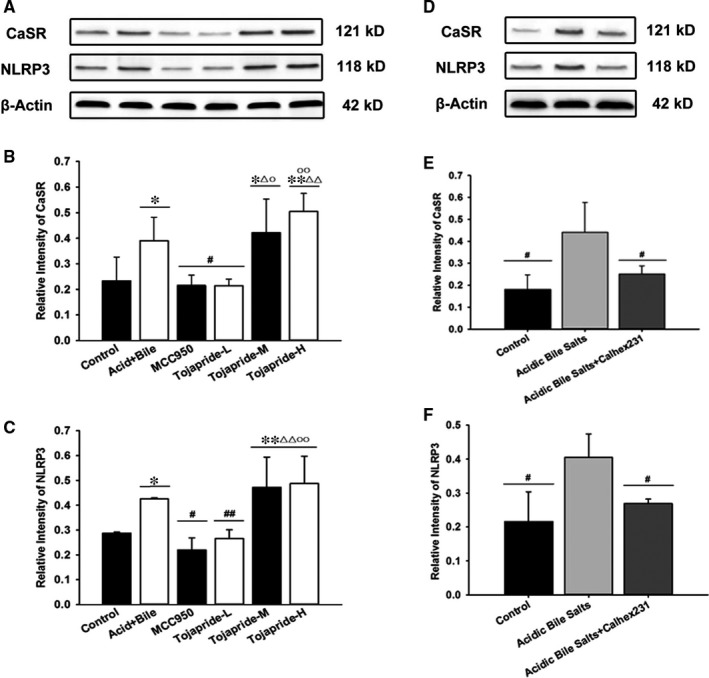
Inhibitory effects of Tojapride on the expression of CaSR‐mediated NLRP3 up‐regulation in HET‐1A cells exposed to acidic bile salts. A‐C, Western blot to investigate the effect of Tojapride on the expression of CaSR and NLRP3 in cells treated with acidic bile salts. D‐F, Western blot showing the effect of 10 μM Calhex231 on the expression of CaSR and NLRP3 in cells treated with acidic bile salts. Bar graphs represent the means ± SD. *P < .05 and **P < .05, compared with the control group; # *P* < .05 and ## *P* < .01, compared with acidic bile salt‐treated group; △*P* < .05 and △△*P* < .01, compared with the MCC950‐treated group; and ○*P* < .05, compared with Tojapride‐L–treated group

To further confirm that the CaSR/NLRP3 inflammasome signalling pathway is up‐regulated in the oesophageal epithelium, which is impaired by mixed refluxate, we investigated the expression of CaSR and NLRP3 in HET‐1A cells stimulated by acidic bile salt treatment. We found that exposure to acidic bile salts significantly increased the expression of CaSR and NLRP3 in HET‐1A cells (Figure 6B,C). In addition, we observed a correlation between the up‐regulation of CaSR and NLRP3 inflammasome activation. Pretreatment of cells with Calhex231, a negative allosteric inhibitor of CaSR, inhibited the expression of both CaSR and NLRP3 after stimulation with acidic bile salts (Figure 6E,F). Tojapride‐L and MCC950 treatment effectively reduced the acidic bile salts‐induced up‐regulation of CaSR and NLRP3 expression, while Tojapride‐M and Tojapride‐H treatment had no such effect (Figure 6B,C).

### 3.7 Exposure of HET‐1A cells to acidic bile salts activates caspase‐1 and causes the secretion of proinflammatory cytokines and release of LDH. Tojapride diminishes the pyroptotic cell lesions through caspase‐1–mediated cytokine and LDH release

**Figure 7:**
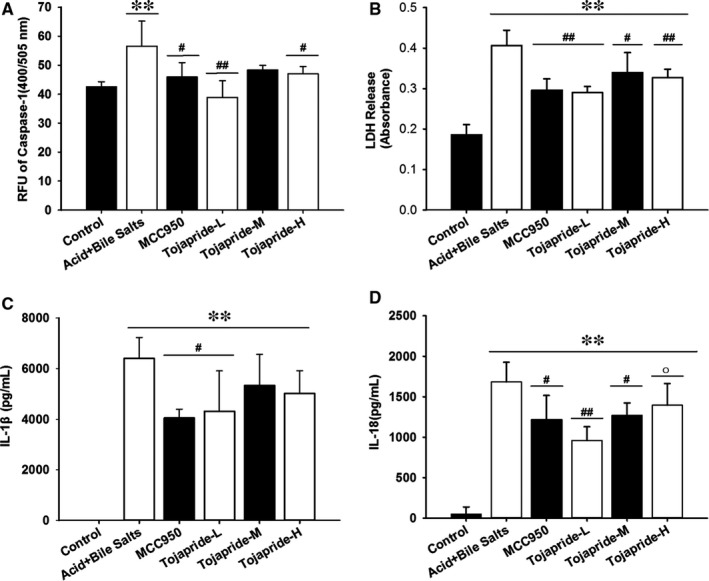
The inhibitory effects of Tojapride on acidic bile salt‐induced caspase‐1 activity increase, LDH release and IL‐1β and IL‐18 secretion. A, Caspase‐1 activity assay. B, LDH release detection. Enzyme‐linked immunosorbent assays for (C) IL‐1β secretion and (D) IL‐18 secretion. Bar graphs represent the means ± SD. *P < .05 and**P < .05, compared with the control group; #*P* < .05 and ##*P* < .01, compared with the acidic bile salt‐treated group; and ○*P* < .05, compared with Tojapride‐L–treated group

The activation of the NLRP3 inflammasome always accompanies caspase‐1–induced pyroptosis, which promotes pore formation and the release of the cytokines IL‐1β and IL‐18. To show that proinflammatory cytokine release and pyroptosis is induced by acidic bile salts, we assessed the protease activity of caspase‐1, the release of LDH (an indicator of pyroptosis) and the concentrations of the secreted cytokines IL‐1β and IL‐18. The results showed that stimulation with acidic bile salts caused a significant increase in caspase‐1 activity, secretion of IL‐1β and IL‐18, and release of LDH; treatment with Tojapride‐L and MCC950 decreased all of the above indicators (Figure 7).

## 4 DISCUSSION

In this study, our exploration of modified RE rats and acidic bile salt‐stimulated HET‐1A cells have yielded several novel findings. The CaSR plays an important role in regulating the differentiation and proliferation of oesophageal epithelial cells, and long‐term activation of CaSR induces cytoskeletal remodelling, actin fibre formation and the redistribution of the CaSR to the nuclear area in oesophageal epithelial cells.^34^ However, Sam X. Cheng et al found that the CaSR‐knockout mice had decreased tight junction protein expression in the colonic epithelium.^7^ Both overexpression and underexpression of CaSR seem to impair the morphology of the gastrointestinal epithelial barrier. In addition to its established function in oesophageal epithelial cell proliferation, some evidence suggests an emerging role for CaSR in modulating inflammation in intestinal mucosa; this research has drawn much attention,^35^, ^36^ but it is unclear whether CaSR functions in the oesophageal epithelium.

CaSR‐mediated NLRP3 activation is thought to lead to the secretion of the proinflammatory cytokine IL‐1β in macrophages. Our present study also found that CaSR‐mediated NLRP3 activation in the oesophagus of modified RE rats led to secretion of the proinflammatory cytokine IL‐1β; however, in contrast to our findings, Sam X. Cheng et al found that CaSR^‐/‐^ mice displayed enhanced intestinal inflammation and immune cell activation through a gut microbe imbalance, finally skewing immunity towards a proinflammatory response. Sam X. Cheng et al suggested that CaSR plays a potentially protective role. Intestinal immune activation in CaSR‐deficient mice might be due to dysbiosis‐induced pathogenic inflammatory immune responses. Gene array analyses showed a marked increase in the expression of a range of PRRs, with the exception of NLRP3.^7^ In support of our findings, Abdulnournakhoul S et al found that the pattern of staining for CaSR was different in different pathological conditions of the human oesophagus, and increased CaSR expression was found in oesophageal biopsies from patients with RE.^35^ Notably, CaSR modulates proliferation in accordance with pathological changes in epithelium and papillary proliferation in patients with RE and induces NLRP3 inflammasome activation causing oesophagitis through a cytokine‐mediated inflammatory response. Furthermore, we found NLRP3‐ASC colocalization in the cross section of whole oesophageal layers, while CaSR expression was limited to the basal zone of the epithelium. To further clarify whether CaSR‐mediated NLRP3 inflammasome activation is the initiating factor of the inflammatory‐cascade response starting from epithelial cells, we used HET‐1A cells stimulated with acidic bile salts to mimic mixed refluxate impairment of the oesophageal epithelium. Our study showed that acidic bile salt treatment reduced the TEER and accelerated CaSR‐mediated NLRP3 up‐regulation, finally activating caspase‐1–mediated pyroptosis and secretion of the cytokines IL‐1β and IL‐18. Nevertheless, the expression of both CaSR and NLRP3 was slightly enhanced in HET‐1A cells stimulated with acidic bile salts, while the secretion of cytokines was markedly increased. We postulated that the activation of the NLRP3 inflammasome is not the only reason for the production of IL‐1β and IL‐18, but one of the proinflammatory pathways modulated by CaSR.

Currently, 8 weeks of continuous PPI therapy remain the mainstay for the management of RE. The healing rate with 8 weeks of esomeprazole treatment was 93.7% and 84.2% with omeprazole therapy.^37^ Although PPI therapy for RE is effective, the side effects of PPI therapy have drawn public attention to this problem. Moreover, the failure of PPI therapy may increase the psychological burden, establishing a vicious cycle.^38^ We found that Tojapride is clinically efficacious against GERD, fulfils unmet needs for PPI therapy and can inhibit CaSR‐mediated NLRP3 inflammasome activation. The Tojapride dosage used in our experiments was converted from the clinical dosage, and we investigated three concentrations of Tojapride to determine the dose‐effect relationship. Our in vivo study clearly demonstrated that Tojapride effectively ameliorated modified RE rat anxiety‐related behaviour, prevented reflux of gastric and duodenal contents, diminished the pathological impairment and inhibited the proinflammatory pathway of the CaSR/NLRP3 inflammasome. There were appreciable differences in the protective effects of Tojapride‐H used throughout the animal experiments, whereas the effect of Tojapride‐L treatment was not different from that of the sham treatment, suggesting that Tojapride might be used at a higher dose to treat patients with RE. Moreover, we found that only Tojapride‐H decreased the expression of CaSR and NLRP3 at both the transcriptional and protein levels and decreased ASC, caspase‐1 and IL‐1β at the protein level, which might contribute to the reduction in the pathological impairment and refluxate contents.

The technique of serum pharmacology used to investigate the TCM formula in vitro is accepted by many scholars of Chinese medicine. We found that MCC950 and Tojapride‐L treatment effectively reversed the reduced TEER and significantly decreased the expression of CaSR and NLRP3. However, Tojapride‐M and Tojapride‐H treatment had no such effect. It is paradoxical that the dose‐effect relationship of Tojapride is contradictory between in vivo and in vitro experiments. Notably, pretreatment with Tojapride‐M and Tojapride‐H significantly up‐regulated CaSR and NLRP3 and increased LDH release and the secretion of proinflammatory cytokines compared with control treatments. The HET‐1A cell line, which exhibits characteristics of normal oesophageal epithelial cells, is nontumorigenic in nude mice and does not induce transient carcinoma‐like nodules at the injection site. The growth of HET‐1A cells is inhibited in serum‐supplemented media because of terminal differentiation, like most epithelial cells.^39^ Even though we removed the proteins from Tojapride serum through acetonitrile and nitrogen blowing, some serum molecules produced as the concentration increased from low to high may have caused increased toxicity after drug biotransformation. We postulate that the protective effect of increased concentrations of the drug serum is surpassed by the cytotoxic effect.

In summary, the current study demonstrates that a formula granule of the TCM Tojapride effectively ameliorates the RE‐related inflammatory response of the oesophageal epithelium through the CaSR/NLRP3 inflammasome pathway. The discovery of the molecular mechanism behind the anti‐inflammatory effect of Tojapride provides insight into the potential application of Tojapride in RE complementary therapy.

## CONFLICT OF INTEREST

The authors declare no competing financial interests.

## AUTHOR'S CONTRIBUTION

Xu‐Dong Tang and Feng‐Yun Wang designed the study; Xiao‐Lan Yin carried out the experiments and draft of the manuscript; Hao‐Meng Wu and Bei‐Huang Zhang co‐participated in draft and publication of the paper. Ting Chen and Xiang‐Xue Ma participated in the experimental design; Li‐Ying Zhang, Lin Lv and Min Zhang coordinated the experiments; all of them helped to conduct the evaluation of TEEM, Western blot and RT‐PCR operation. Ning‐Wei Zhu involved in discussion of experiments. All authors read and approved the final manuscript.
